# Connecting Cancer Pathways to Tumor Engines: A Stratification Tool for Colorectal Cancer Combining Human In Vitro Tissue Models with Boolean In Silico Models

**DOI:** 10.3390/cancers12010028

**Published:** 2019-12-20

**Authors:** Florentin Baur, Sarah L. Nietzer, Meik Kunz, Fabian Saal, Julian Jeromin, Stephanie Matschos, Michael Linnebacher, Heike Walles, Thomas Dandekar, Gudrun Dandekar

**Affiliations:** 1 Chair of Tissue Engineering and Regenerative Medicine, University Hospital Würzburg, Röntgenring 11, 97070 Würzburg, Germany; baur@em.uni-frankfurt.de (F.B.); sarah.nietzer@uni-wuerzburg.de (S.L.N.); heike.walles@ovgu.de (H.W.); 2 Fraunhofer Institute for Silicate Research (ISC), Translational Center Regenerative Therapies, Röntgenring 11, 97070 Würzburg, Germany; 3 Chair of Medical Informatics, Friedrich-Alexander University of Erlangen-Nürnberg, 91058 Erlangen, Germany; meik.kunz@fau.de; 4 Department of Bioinformatics, Biocenter, University of Würzburg, Am Hubland, 97074 Würzburg, Germany; fabian.saal@googlemail.com (F.S.); julian.jeromin@stud-mail.uni-wuerzburg.de (J.J.); 5 Department of Surgery, Molecular Oncology and Immunotherapy, University Medical Center Rostock, Schillingallee 35, 18057 Rostock, Germany; stephanie.matschos@med.uni-rostock.de (S.M.); michael.linnebacher@med.uni-rostock.de (M.L.); 6 EMBL Heidelberg, Structural and Computational Biology, Meyerhofstraße 1, 69117 Heidelberg, Germany

**Keywords:** in silico simulation, 3D tissue models, colorectal cancer, BRAF mutation, targeted therapy, stratification

## Abstract

To improve and focus preclinical testing, we combine tumor models based on a decellularized tissue matrix with bioinformatics to stratify tumors according to stage-specific mutations that are linked to central cancer pathways. We generated tissue models with *BRAF*-mutant colorectal cancer (CRC) cells (HROC24 and HROC87) and compared treatment responses to two-dimensional (2D) cultures and xenografts. As the BRAF inhibitor vemurafenib is—in contrast to melanoma—not effective in CRC, we combined it with the EGFR inhibitor gefitinib. In general, our 3D models showed higher chemoresistance and in contrast to 2D a more active HGFR after gefitinib and combination-therapy. In xenograft models murine HGF could not activate the human HGFR, stressing the importance of the human microenvironment. In order to stratify patient groups for targeted treatment options in CRC, an in silico topology with different stages including mutations and changes in common signaling pathways was developed. We applied the established topology for in silico simulations to predict new therapeutic options for BRAF-mutated CRC patients in advanced stages. Our in silico tool connects genome information with a deeper understanding of tumor engines in clinically relevant signaling networks which goes beyond the consideration of single drivers to improve CRC patient stratification.

## 1. Introduction

Colorectal cancer (CRC) is the third most common cancer worldwide in men, second in women, and 55% of the cases occur in developed countries [1]. About 15% of CRCs develop as a consequence of microsatellite instability (MSI) and therefore represent a specific subgroup of tumors. Recent analysis of sequence data from over 9000 tumor samples of “*The Cancer Genome Atlas*” demonstrated that genetic alterations concentrate in 10 common signaling pathways composing over 30 tumor types. CRC with MSI showed high alteration rates in nearly all of these pathways with most changes occurring in the mitogen-activated protein kinase (MAPK) and Wnt/β-catenin signaling pathways [2].

The activating BRAF missense mutation V600E is part of the MAPK pathway and occurs in 59% of melanoma patients and in at least 10% of CRC [3] being a powerful prognostic factor [4,5]. In CRC, the frequency of BRAF V600E mutation is 78% for MSI tumors as compared to only 8% in tumors with chromosomal instability pathway (CIP) [6]. The selective FDA-approved BRAF inhibitor vemurafenib (PLX4032), achieves an impressive clinical response in melanoma [7,8], but fails in most CRCs [9,10]. This may be due to a feedback activation of the epidermal growth factor receptor (EGFR) as its additional inhibition by the small molecule gefitinib (Iressa^®^) improved suppression of the MAPK signaling pathway in vitro [11,12]. The combination-therapy of BRAF and EGFR inhibition showed first but not yet fully conclusive success [13,14,15]. This stresses the urgent need for a deeper understanding of dependencies in common cancer pathways. To achieve this in a mathematical way, we pursue Boolean models that represent in silico the “yes or no logic” of tumor associated pathways [2] and the corresponding mutational regulation [3,4,5]. Moreover, the “switch on and off” of central connected driving forces of the tumor—the engines— [2] and the complex balances dominating cellular decision processes regarding proliferation, apoptosis, inflammation or growth arrest can be integrated within this approach. Boolean models have proven to reflect biological systems [16,17]. We use the topology of relevant signaling pathways to set up a Boolean interaction network as calculation base for refined new dynamic models allowing more quantitative predictions. For example, these models reveal the extent of proliferation or apoptosis without detailed knowledge of the involved kinetics of individual kinases and other molecular rate constants [18,19].

Commercially available cell lines grown under conventional two-dimensional (2D) in vitro cell culture conditions often do not reflect important characteristics of the tumor [20,21]. However, the in vivo situation relies on a 3D microenvironment that alters cell morphology and organization, proliferation, signaling, differentiation, gene expression and cell surface receptors, generally leading to a higher drug resistance compared to 2D conditions [22,23,24,25,26]. We used MSI CRC cell lines directly derived from patient-derived xenografts (PDX) [27]. These cell lines, HROC24 and HROC87 (Hansestadt ROstock—Colon), harbor a BRAF mutation with further co-mutations: HROC24 with APC mutation and HROC87 with p53 mutation [27,28,29]. For our CRC models, we use an avascular part of a decellularized porcine gut [30,31,32,33]—called small intestine submucosa with preserved mucosa (SISmuc) [34], that is also applied for lung cancer and breast cancer models [18,19,35,36,37]. The preserved basal membrane structure enables physiological anchorage of epithelial cells and the analysis of invasive processes. In lung cancer, we observed better predictivity compared to 2D as we could, for example, mirror the clinical attrition of an anti-HSP90 therapy in patients with a KRAS mutation that was suggested by other preclinical models [19,35]. Moreover, these 3D models were combined with an in silico tool for the development of signaling- and mutational-based signatures for therapy predictions in stratified patient groups [18,35].

In this study, we created CRC tissue models and an in silico topology integrating database information and scientific literature to simulate the transformation of healthy colon epithelial cells to a metastasizing carcinoma including critical mutations, interconnected subnetworks [38], processes such as the epithelial to mesenchymal transition (EMT), and different cellular responses. The in silico topology was adapted to experimentally measured differences in proliferation, apoptosis, and cell signaling upon drug treatment. Subsequently, we used this topology to predict individualized anticancer drug response. This therapeutic strategy provides insights into general mechanisms that can be exploited for better patient stratification in CRC, extending mutational profiles to functional tumor stages.

## 2. Results

### 2.1. Characterization of Low Passage Cell Lines and Comparison of 3D Tissue Morphology with Cancerous Specimens

**Figure 1:**
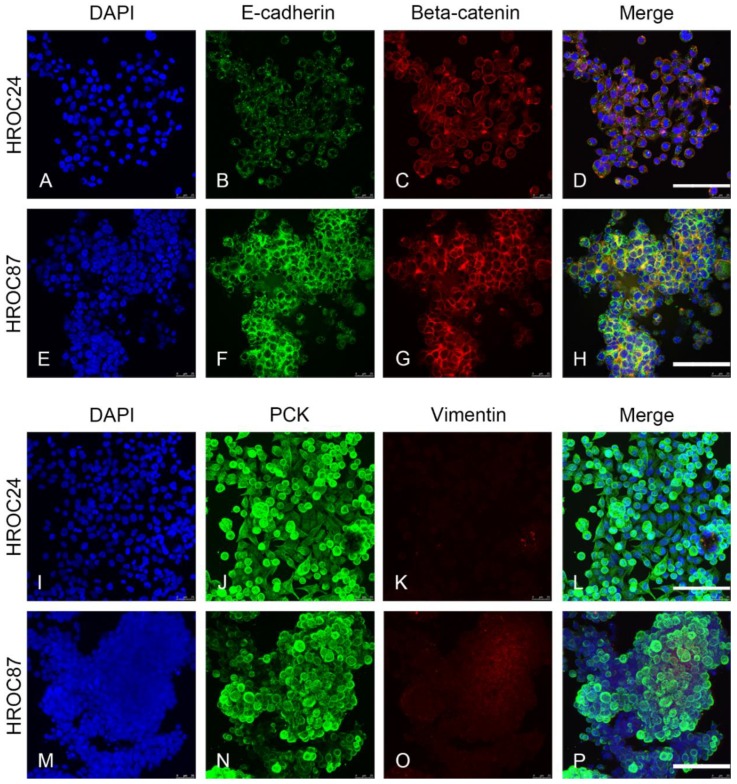
Characterization of 2D-cultured HROC24 and HROC87 cells. Both cell lines express E-cadherin ((**B**,**F**), green in (**D**,**H**)) and β-catenin ((**C**,**G**), red in (**D**,**H**)) as well as PCK ((**J**,**N**), green in (**L**,**P**)), but no vimentin ((**K**,**O**), red in (**L**,**P**)). E-cadherin and β-catenin clearly co-localize at the cell-to-cell borders in HROC87 tumor cells (yellow in (**H**)). Cell nuclei were counterstained with DAPI (**A**,**E**,**I**,**M**). Scale bars in (**D**,**H**,**L**,**P**): 75 µm for (**A**–**P**).

**Figure 2:**
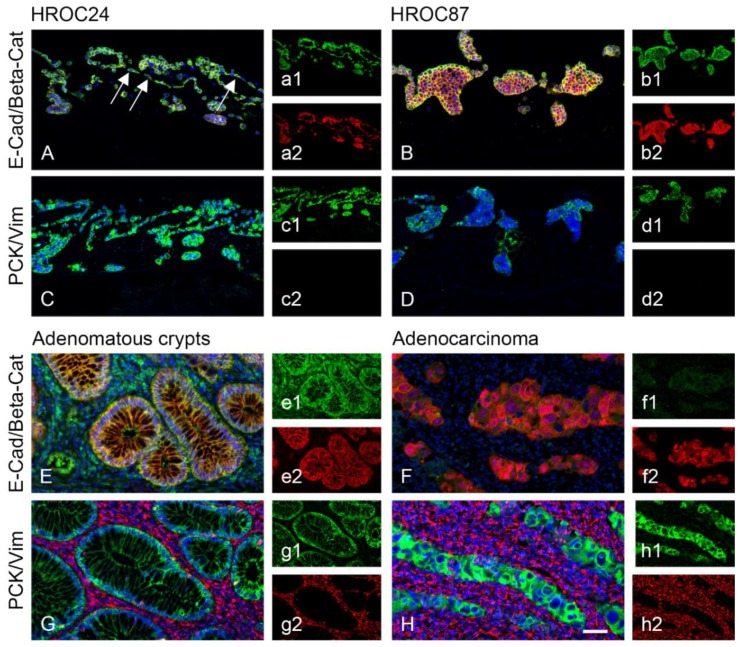
Characterization of the cell lines HROC24 as well as HROC87 cultured on the scaffold SISmuc. While HROC24 cells grow in monolayers and fill the former crypts of the gut (**A**,**C**), HROC87 cells grow in dense clusters (**B**,**D**). E-cadherin is highly expressed at the cell-to-cell contacts of HROC24 cells (green in (**A**,**a1**)) as well as HROC87 cells (green in (**B**,**b1**)). β-catenin is more strongly expressed in HROC87 cells (red in (**B**,**b2**)) than in HROC24 cells (red in (**A**,**a2**)). Both cell lines are positive for PCK (green in (**C**,**D**), (**c1**,**d1**)) and do not express vimentin (**c2**,**d2**). For comparison, adenomatous crypts as well as adenocarcinoma tissue were stained with E-cadherin (green in (**E**,**F**), (**e1**,**f1**)), β-catenin (red in (**E**,**F**), (**e2**,**f2**)), PCK (green in (**G**,**H**), (**g1**,**h1**)) and vimentin (red in (**G**,**H**), (**g2**,**h2**)). Scale bar in (**H**): 50 µm for (**A**–**H**).

**Figure 3:**
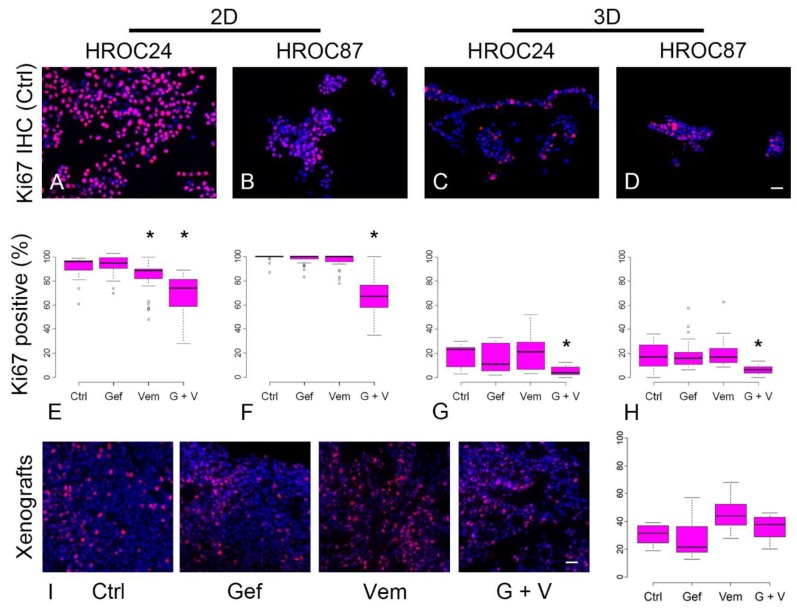
Proliferation of HROC24 and HROC87 cells is influenced differentially by vemurafenib, gefitinib or the combination-therapy. (**A**–**D**): Ki67 (red) and DAPI (blue) staining of cells in 2D (**A**,**B**) and 3D (**C**,**D**) cell culture. In 3D, proliferation is reduced compared to 2D. While under 2D culture conditions, HROC24 cells respond to vemurafenib and to the combination-therapy by reduced proliferation (**E**), HROC87 cells respond only to the combination-therapy (**F**). Cultured in 3D, proliferation is in both cell lines only reduced by the combination-therapy (**G**,**H**). PDX of HROC87 were stained with Ki67/DAPI and proliferation was evaluated (**I**). No changes were detected after different treatments in these PDX. *: *p* < 0.05. Ctrl: untreated control, Gef = gefitinib, Vem = vemurafenib, G + V = combination-therapy with gefitinib and vemurafenib. Scale bars in D and I: 50 µm for A to D and I. ° denotes outliers.

For 3D models, HROC24 and HROC87 cell lines were seeded onto the SISmuc scaffold derived from porcine gut and cultured for 14 days. To assess malignancy, EMT status of the cells was evaluated by immunohistochemical staining of E-cadherin, β -catenin, Pan-cytokeratin (PCK) and vimentin in 2D (Figure 1) and 3D (Figure 2).

**Figure A1:**
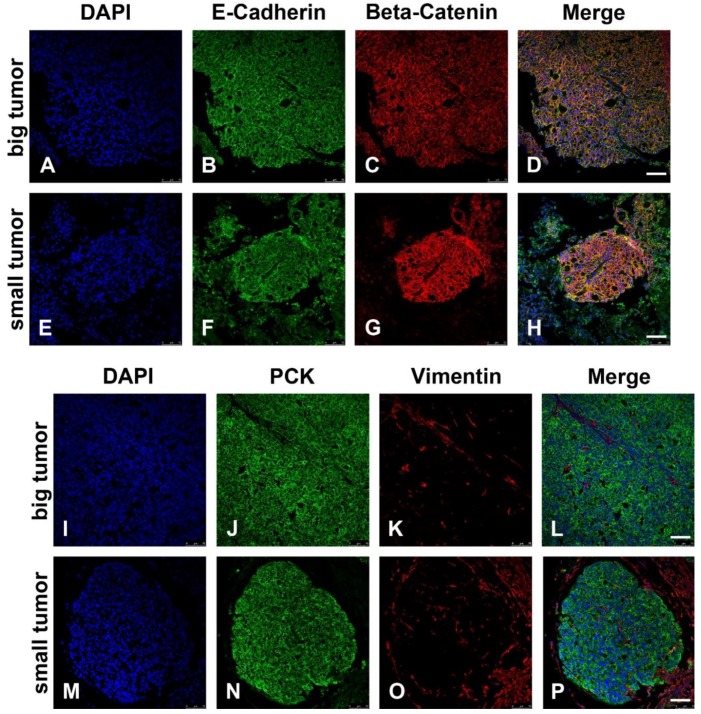
HROC87 PDX characterization. E-cadherin is highly expressed at the cell-to-cell contacts of HROC87 cells (green in (**B**,**D**,**F**,**H**)) in the xenografts. β -catenin is highly expressed in HROC87 cells (red in (**C**,**D**,**G**,**H**)). Cells are positive for PCK (green in (**J**,**L**,**N**,**P**)) and the tumor cells do not express vimentin while the murine stroma is stained (red in (**K**,**L**,**O**,**P**)). Scale bar in (**D**,**H**,**L**,**P**): 50 µm for (**A**–**P**).

**Figure A2:**
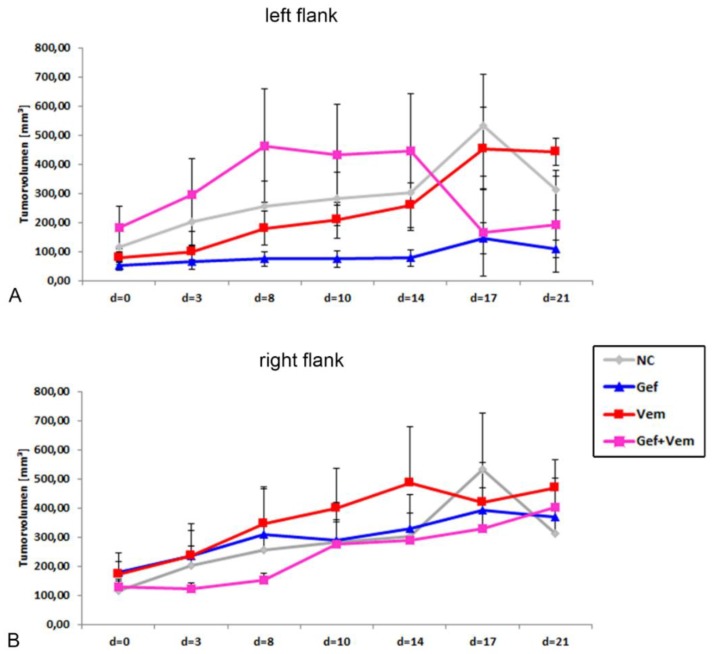
HROC87 PDX volumes. Volumes of HROC87 xenografts do not differ between the various treatment conditions. Moreover, there is considerable variation and hence in these animal experiments treatment differences do not really become apparent. This was the case in the left (**A**) as well as the right (**B**) flank. NC: untreated control, Gef: gefitinib, Vem: vemurafenib, Gef + Vem: combination-therapy with gefitinib and vemurafenib.

**Figure A3:**
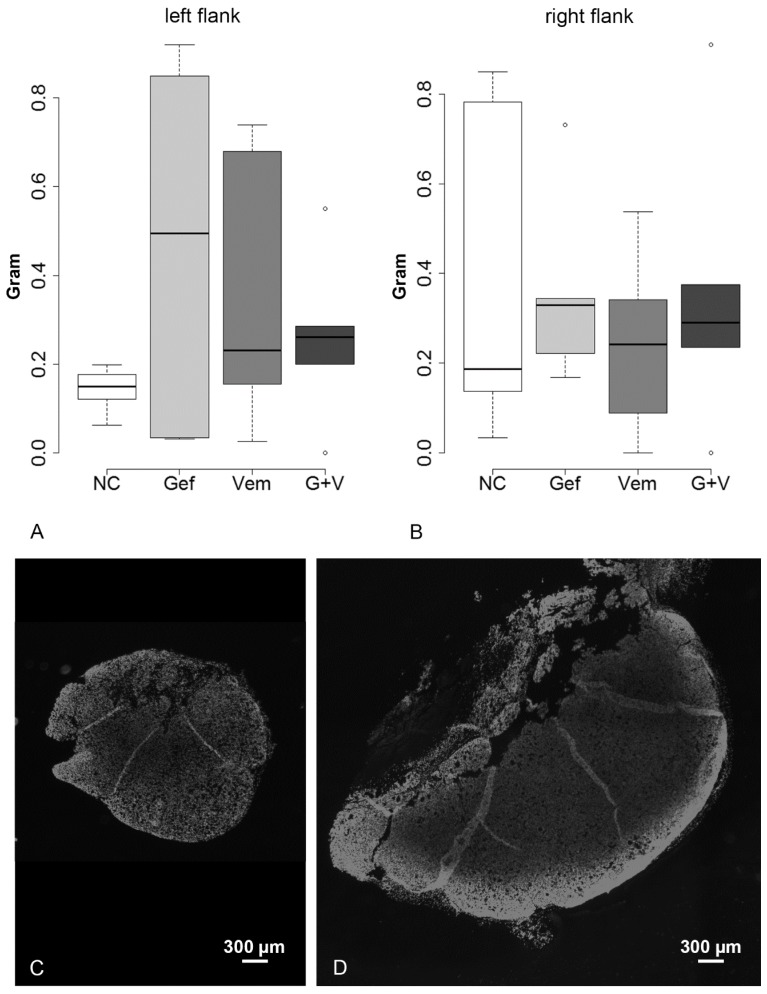
Tumor weight difference of different tumor groups. Tumor weights were very variable within the different treatment groups in the left (**A**) as well as in the right (**B**) flanks. Tumor size as well as weight could be different in the two tumors of the same mouse (**C**,**D**). Again, with this variation in animal experiments treatment differences do not really become apparent.

In 2D and 3D, all cells were clearly epithelial (positive for E-cadherin, β-catenin and PCK, negative for vimentin). In 3D, HROC24 cells built a monolayer on the surface (arrows in Figure 2A) of the SISmuc and filled single crypts with cells. In contrast, HROC87 cells formed no monolayer but tight cell aggregates on the matrix surface and grew only in some of the former crypts of the intestine (Figure 2B). Both cell lines thereby nicely reflect the differentiation state of their tumors of origin. β -catenin staining was stronger in HROC87 than in HROC24 cells but remained localized at the cell borders. PDX of HROC87 showed the same expression of all markers (Figure A1) and were of different size (Figure A2) and weight (Figure A3), independent of treatment conditions.

In comparison to patient specimens, our 3D models from HROC24 cells morphologically resembled adenomatous crypts, HROC87 cells the more advanced stage of an adenocarcinoma. Adenomatous crypts expressed E-cadherin at the cell-to-cell contacts of the crypts colocalized with β-catenin (Figure 2E). In an adenocarcinoma, E-cadherin expression was reduced and β -catenin was localized in the cytoplasm and also in the nuclei of the specimen (Figure 2F). As HROC87 cells derive from an adenocarcinoma without β-Catenin translocation [27], our model reflects the original condition in the patient. Both tumors showed no vimentin expression similar to the in vitro results.

Next, we were interested in treatment responses of the different models. Therefore, we compared the effects of the BRAF inhibitor vemurafenib when applied to 2D- and 3D-cultured cells and to PDX as monotherapy and in combination with the EGFR inhibitor gefitinib. To link these results to common pathways, we measured not only cell survival rates, but also proliferation and apoptosis as these responses emerge from different signaling cascades.

### 2.2. Proliferation and Apoptosis Responses upon Treatment

We assessed proliferation in 2D and 3D cell cultures by counting Ki67-stained cells and observed in general a lower proliferation in 3D than in 2D (Figure 3A–H).

Proliferation was significantly decreased by vemurafenib and the combination-therapy in 2D-cultured HROC24 cells (Figure 3E), whereas proliferation of HROC87 cells was only significantly decreased by combining vemurafenib and gefitinib (Figure 3F). In 3D, HROC24 cells and HROC87 cells both showed significantly reduced proliferation rates only after combination-therapy (Figure 3G, H). Proliferation rates of HROC87 PDX were not influenced by different treatments (Figure 3I).

**Figure 4:**
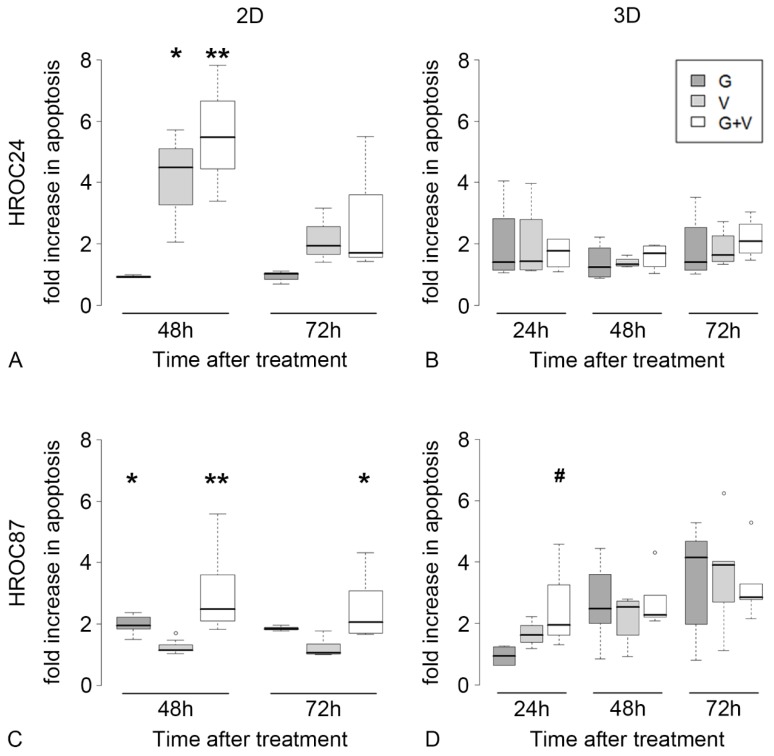
Apoptosis rates in HROC24 versus HROC27 cells in 2D and 3D. We compared apoptosis rates (fold increase to untreated controls) of gefitinib treatment (G), vemurafenib (V) or their combination (G + V). HROC24 cells respond to monotherapy with vemurafenib in 2D as well as to the combination-therapy (**A**). In contrast, HROC87 cells respond to gefitinib monotherapy in 2D as well as to the combination-therapy (**C**). In 3D, both drugs are not able to significantly induce apoptosis, neither in monotherapy nor in combination-therapy in HROC24 (**B**) and in HROC87 cells (**D**). #: 0.05 < *p* < 0.1, *: 0.01 < *p* < 0.05, **: *p* < 0.01. ° denotes outliers.

In 2D, the monotherapy with vemurafenib and the combination-therapy significantly increased the apoptosis rate in HROC24 cells after 48h (Figure 4A).

HROC87 cells responded significantly to gefitinib and the combination-therapy by increased apoptosis (Figure 4C). In contrast, in 3D we observed only a trend but no significant apoptosis induction in HROC24 (Figure 4B) and HROC87 cells (Figure 4D).

### 2.3. Comparison of Signaling Changes upon Treatment between Different Models

**Figure 5:**
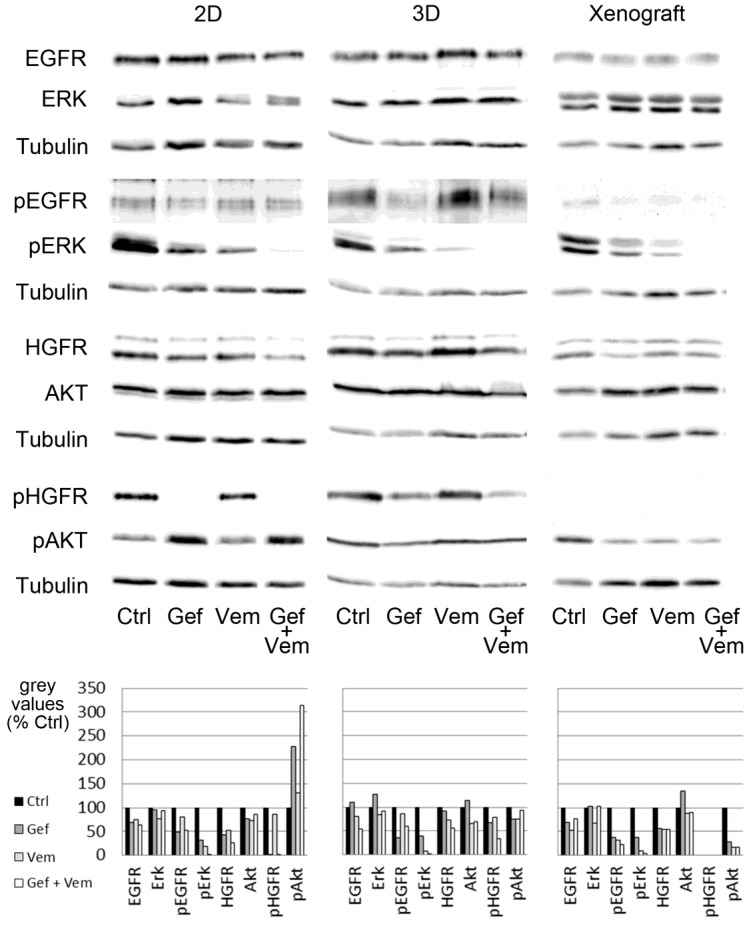
Signaling in HROC87 cells upon treatment with vemurafenib and/or gefitinib. Shown is the Western blot analysis of the phosphorylation status of molecules involved in signal transduction for proliferation (ERK) and apoptosis (AKT) and important receptors in HROC87 cells cultured under 2D and 3D conditions as well as in matching xenografts. In 2D, 3D and PDX, ERK is partly inhibited by gefitinib and vemurafenib in monotherapy, but nearly completely inhibited by the combination-therapy. In contrast to 2D, AKT phosphorylation show no clear upregulation under 3D culture conditions. HGFR could not be activated in the PDX at all. Only in the 2D model, gefitinib and the combination-therapy results in a strong inactivation of the HGFR. Ctrl: untreated control, Gef: gefitinib, Vem: vemurafenib, Gef + Vem: combination-therapy of gefitinib and vemurafenib. Grey values of densitometric analysis of the representative blots are shown in the diagrams (% of Ctrl). Blots with weight markers are shown in Figure A4, Figure A5 and Figure A6. Densitometric analyses of grey values from all 4 to 6 experiments (Figure A7) as well as from the representative blot depicted here (Table A1) are shown in the appendix.

**Figure A4:**
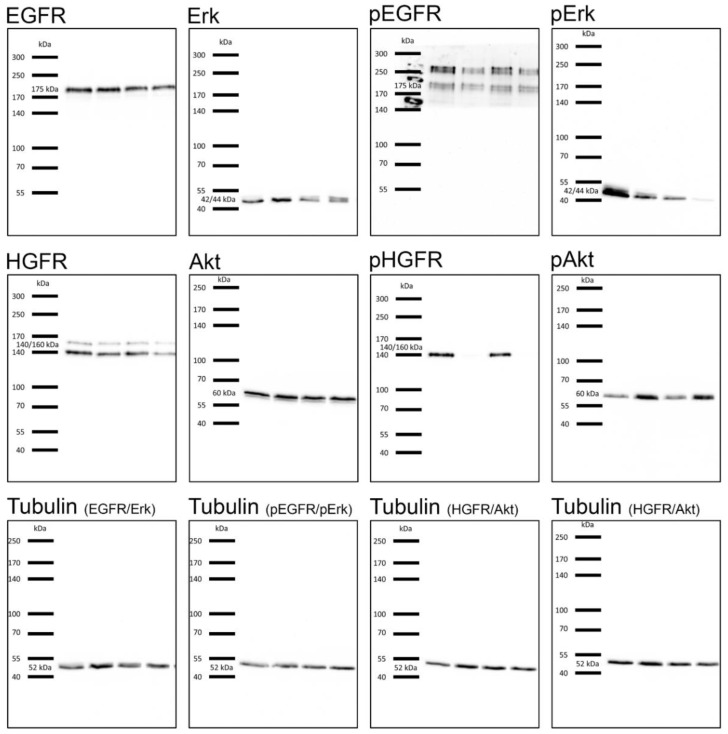
Blots with molecular weight marker of the 2D experiments shown in Figure 5.

**Figure A5:**
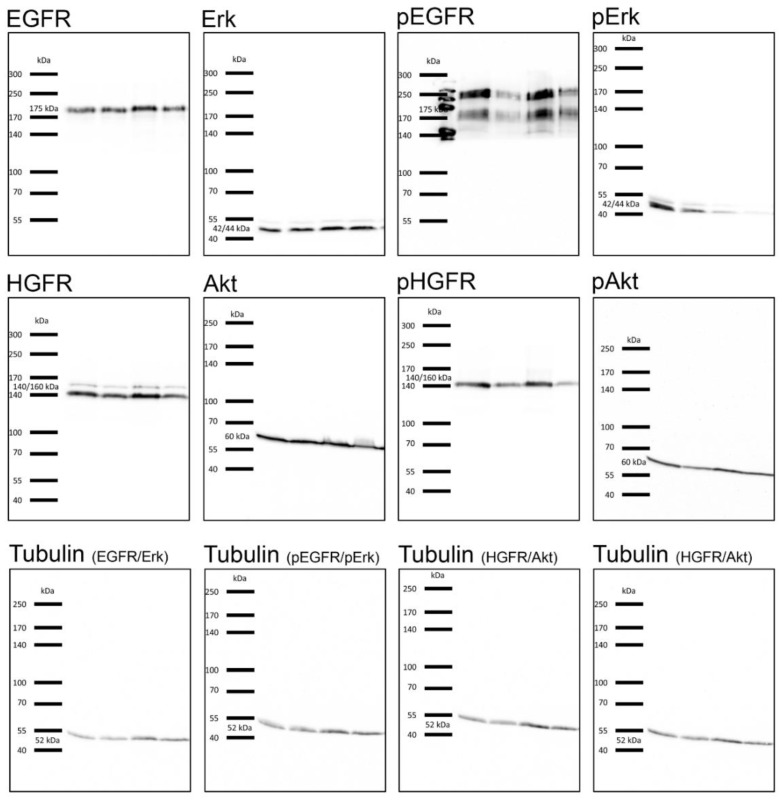
Blots with molecular weight marker of the 3D experiments shown in Figure 5.

**Figure A6:**
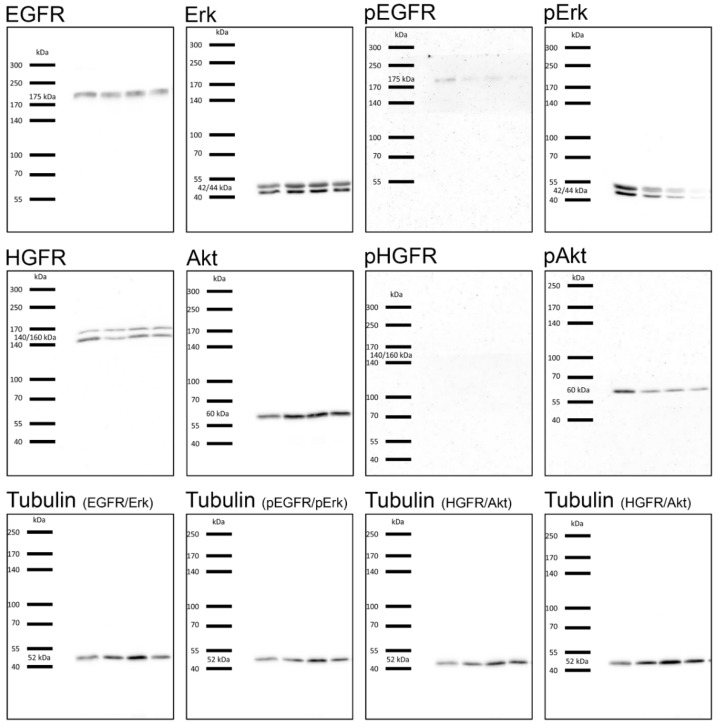
Blots with molecular weight marker of the PDX experiments shown in Figure 5.

**Figure A7:**
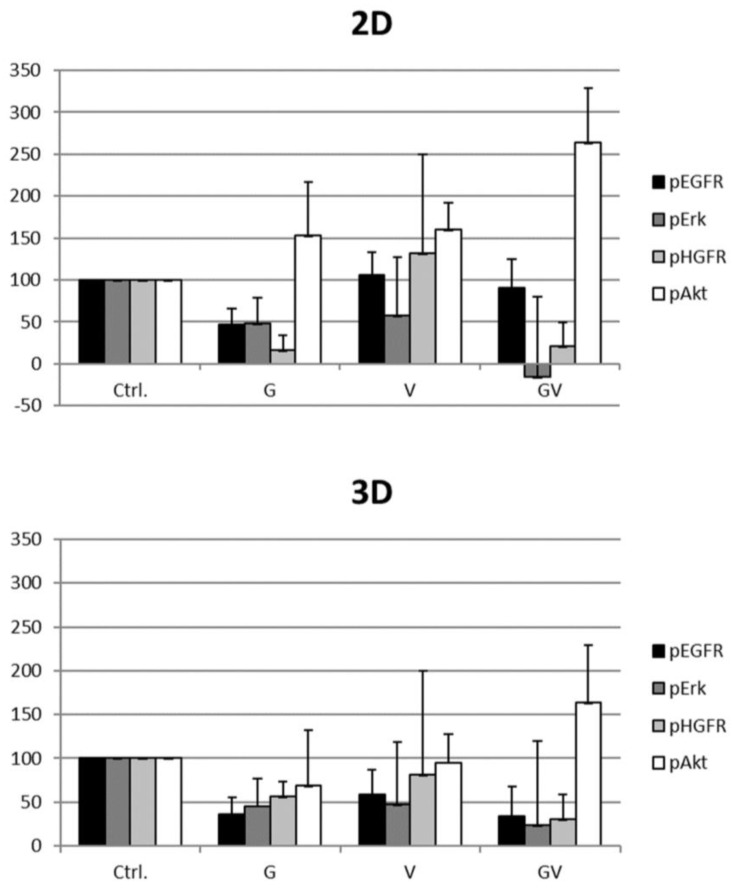
Grey values (in % of control) of 4–6 western blots. For 2D, the values of 4–5 western blots and for 3D, the values of 5–6 western blots were analyzed. Bars: arithmetic mean. Error bars: standard deviation. Ctrl = untreated control, G = gefitinib, V = vemurafenib, GV = combination-therapy of gefitinib and vemurafenib. pEGFR = phospho-EGFR, pErk = phospho-Erk, pHGFR = phospho-HGFR, pAkt = phospho-Akt.

Next, we analyzed if in signaling cascades centrally involved regulators of apoptosis and proliferation differ between the applied in vitro models (Figure 5; original blots in Figure A4, Figure A5 and Figure A6, densitometry analysis in Table A1, Figure A7).

**Figure 6:**
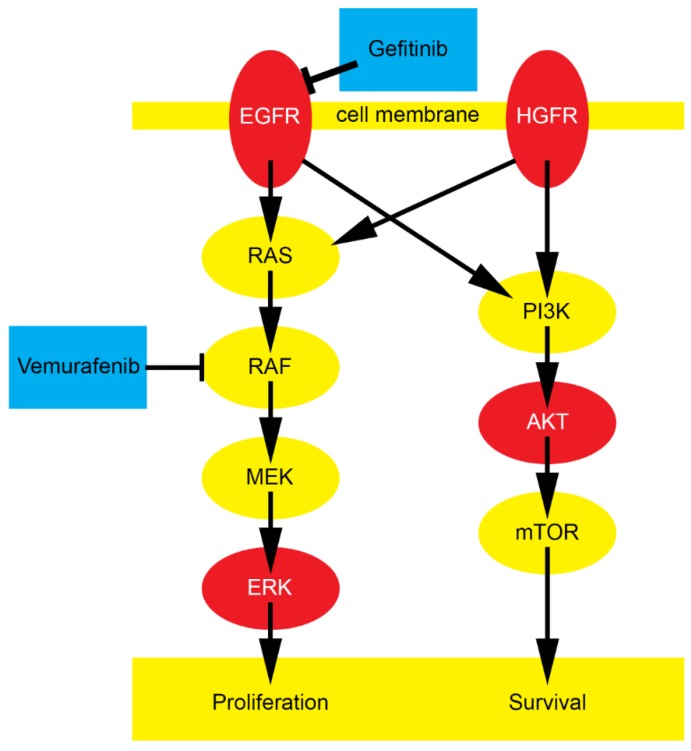
Simplified overview of central pathways involved in proliferation and survival in CRC. EGFR is inhibited by gefitinib, but there is an interference with HGFR. While vemurafenib only blocks the left pathway in case of BRAF mutation, this interplay between the two pathways could be a reason for the low efficacy of vemurafenib in CRC even in case of an existing BRAF mutation.

We focused on HROC87 cells because these cells derive from a more advanced tumor stage. Beside the two receptors EGFR and HGFR we investigated ERK, a protein downstream of BRAF at the end of the MAPK cascade which is a main regulator of proliferation, and AKT (Ser473), one of the most important components regulating cell survival and apoptosis [39]. A simplified overview of these central pathways is given in Figure 6.

All three model types (2D, 3D and PDX) showed decreased ERK phosphorylation after addition of gefitinib or vemurafenib that further diminished after the co-treatment (Figure 5, Table A1 and Figure A7).

We observed AKT activation in 2D after all treatments being strongest after combination-therapy (Figure 5, Table A1 and Figure A7). Contradictory, we measured in 2D conditions significant apoptosis induction upon gefitinib therapy and the combination-therapy. Under 3D conditions, AKT was active after all treatments, but the densitometric evaluation of all experiments revealed a slight reduction in activity upon gefitinib treatment and a tendency to be activated in response to the combination-therapy (Figure A7). Consistently, we observed no significant induction of apoptosis in 3D. In the PDX no strong differences were found in AKT phosphorylation in comparison of the different treatment conditions. 

We also assessed the activation level of the EGFR as a target of gefitinib and the activation level of HGFR as an additional important tyrosine kinase receptor. In 2D, EGFR phosphorylation (pEGFR) decreased after gefitinib treatment. This effect was slightly stronger in 3D than in 2D and could only be seen to some extent in the PDX. Regarding vemurafenib, we observed no clear increase of pEGFR after treatment in 2D and 3D (Table A1 and Figure A7). Notably, we could not detect any activation of the HGFR in the animal model. In comparison to 2D culture, the HGFR was more active in 3D after gefitinib treatment and the combination-therapy, thus underlining the higher chemoresistance of the 3D approach.

### 2.4. In silico System Responses Reflecting 3D In Vitro Data by Integrating HROC87 Specific Mutations and Signaling Cascades

**Figure A8:**
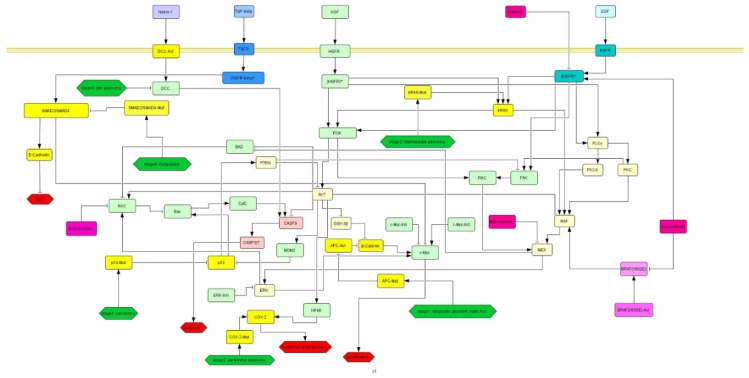
General idealized topology for CRC. The topology includes several relevant signaling cascades shown in different colors. Mutations that lead to cancer progression are marked in yellow, corresponding stages as green hexagons. Possible drugs applied in this study for in silico modeling are added in pink. Cellular responses such as apoptosis and proliferation are indicated in red. Activation of nodes (proteins or systemic responses) is illustrated by arrows, inhibition by blunted arrows.

To gain a deeper understanding of common cancer pathways and interdependencies during tumorigenesis, we created an in silico topology based on information from databases and scientific literature to encapsulate relevant pathways in silico [38,39]. The resulting topology integrates centrally involved proteins of the common signaling pathways and generally accepted cancer drivers in CRC—such as APC, p53m, KRAS, DCC, TGFβR, PTEN and SMAD4 allowing a simplified view on the progression from normal cells to tumor cells (Table A2, Figure A8) [2,40,41,42].

**Figure 7:**
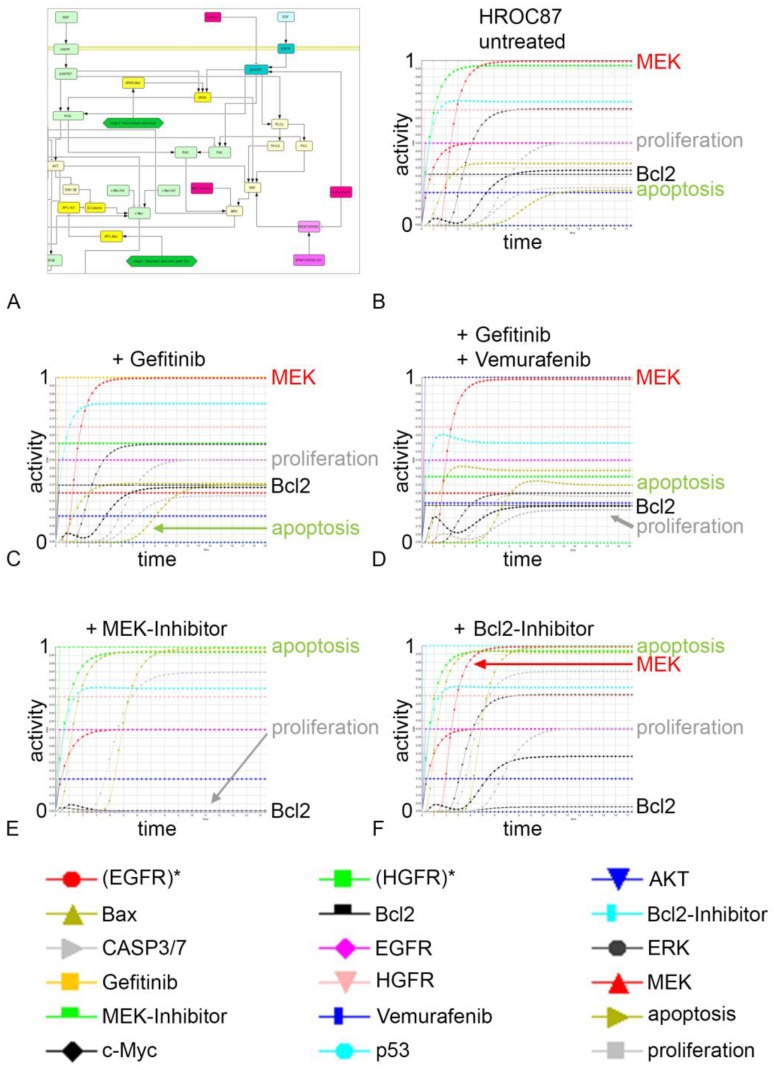
In silico modeling of drug-responses of in 3D cultured HROC87 cells. (**A**) Part of the topology (the complete network is given in Figure A8 of the appendix). Our in silico topology accommodates well-documented major growth and apoptotic cascades in CRC and mimics as close as possible the experimental treatment responses as well as the behavior regarding untreated growth. Directed arrows in the network topology represent activation while blunted arrows represent inhibition. Rectangles: genes/proteins. (**B**) Untreated condition. HROC87 cells show a malignant phenotype with a weak activation of apoptosis (olive-green triangle curve) and high proliferation (grey square curve) signal. Visible in the different curves is also the EGFR (EGFR*), the HGFR (HGFR*), ERK, MEK and additional relevant curves such as Casp3/7. (**C**) Gefitinib treatment. HROC87 cells show only a weak induction of apoptosis (olive-green triangle curve), but no changes in proliferation (grey square curve) upon gefitinib (yellow square curve, activation: 1). (**D**) Combination treatment (gefitinib plus vemurafenib). HROC87 cells show only a weak induction of apoptosis (olive-green triangle curve) and reduction of proliferation (grey square curve). Treatments: gefitinib (yellow square curve, activation: 1, not seen because hidden by the curve for vemurafenib) and vemurafenib (blue rectangle curve, activation: 1). (**E**) MEK inhibition. This shows an induction of apoptosis (olive-green triangle curve) and reduction of proliferation (grey square curve) in HROC87 cells. Notably, there is an inactivation of ERK and Bcl2. (**F**) Bcl2 inhibition. As expected from blocking an anti-apoptotic protein (Bcl2), the simulation suggests an induction of apoptosis (olive-green triangle curve, positive control) but proliferation stays unchanged as there is no connection with MEK-ERK. * = activated form.

A central view of signaling cascades and nodes of the network connecting MAPK with BRAF and vemurafenib is given in Figure 7A, the full network is in the Appendix (Figure A8). For in silico modeling, we considered the HROC87 mutations p53 and BRAF as well as the experimentally measured changes in activation of EGFR, HGFR, ERK and AKT upon different therapies for data-driven modeling using the Standardized QUAlitative Dynamical Modeling (SQUAD) software (Table A3).

This required manually iterative refinement of the in silico model, until the activation level of key signaling nodes as well as proliferation and apoptosis of in vitro models are correctly mirrored. The pre-simulation values of our in silico model are given in Table 1.

These pre-simulation values represent the activation levels of the mutations, experimental nodes and associated signaling pathway nodes to start the simulations, subsequently leading to the different treatment outcomes in silico (Table 2), reflecting results of the in vitro model.

**Figure A9:**
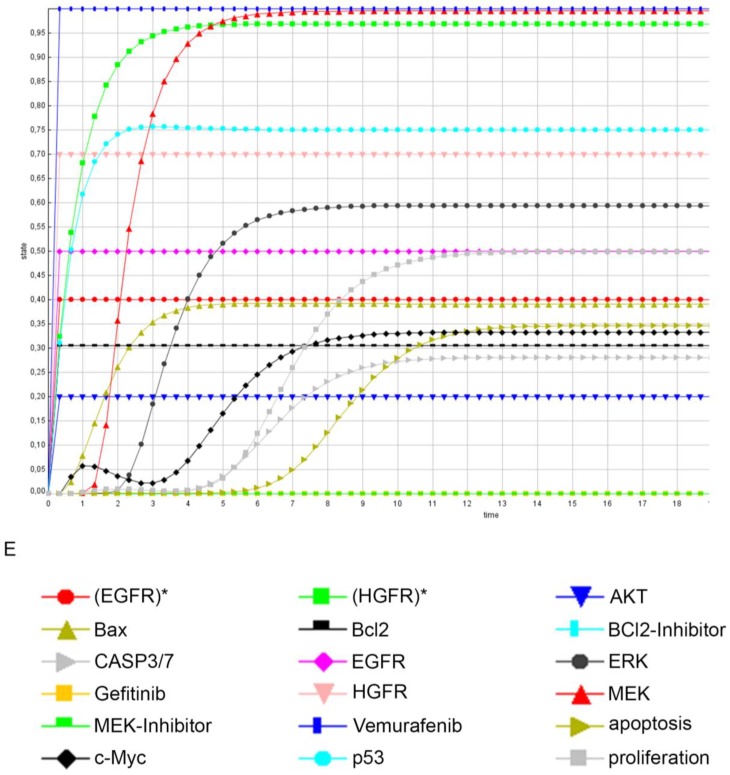
Simulation of vemurafenib treatment of HROC87 cells. The cells show in 3D culture only a weak induction of apoptosis (olive-green triangle curve) but no changes on proliferation (grey square curve) upon vemurafenib (blue rectangle curve, activation: 1). * = activated form.

This is done in a simplified way by considering specific activation levels according to the experimental data as we only model a subnetwork of the complete cancer cell. The software SQUAD [43] models semi-quantitatively the cellular network dynamics of receptor activation, signal processing and cellular output including selective inhibition/activation of individual proteins by drugs to predict tumor-specific responses. The results of dynamical therapy simulations including changes of every node of interest in the network was visualized graphically (Figure 7B–D). In our example, we simulated the untreated situation (Figure 7B), the inhibition of EGFR by gefitinib (Figure 7C), the inhibition of BRAF by vemurafenib (Figure A9) and their combination (Figure 7D). However, even the combination-therapy was not completely effective in our simulations reflecting thereby the results of 3D models and also clinical results. As possible effective targets our simulation suggests MEK and Bcl2, because these stay upregulated over time (red and black lines in Figure 7B–D).

### 2.5. Breaking Resistance: In Silico Mode-of-Action Analysis in p53 and BRAF Mutation Background

We established our combined in vitro/in silico model with the vision to translate individual genomic mutational profiles into predictions of targeted therapy efficacy in order to prevent therapy failure and to help the patients in case of therapy resistances.

As induction of apoptosis is often hindered in therapy-resistant tumor cells, but is important for an effective therapy, we sought how to connect relevant nodes of the in silico topology to the known apoptosis pathways. We experimentally observed major differences between the 2D and 3D models concerning HGFR and AKT activation upon different therapies together with significant apoptosis induction exclusively in 2D but not in 3D.

Anti-apoptotic Bcl2 is activated by ERK and AKT. Next to RAS and RAF, HGFR activates also AKT, which explains why Bcl2 could still be activated by AKT when ERK is strongly downregulated as shown in the Western Blot (Figure 5) resulting in a weak apoptotic effect in the 3D system and illustrated by the simulations in Figure 7B–D. However, P53 activates apoptosis via BAX (inhibited by Bcl2) and PTEN (inhibits AKT). In the patient group with a p53 mutation, this tumor suppressor does not activate the phosphatase PTEN anymore so that AKT stays active as also experimentally observed and apoptosis is inhibited. Furthermore, MEK and AKT inhibit pro-apoptotic BAD, in which the latter one inhibits Bcl2. This could further explain that we can see no strong apoptosis induction in the 3D system after treatment due to the missing inhibition of Bcl2 via BAD.

The cell line HROC87 harbors also a BRAF mutation. Beside apoptosis, the RAS-RAF-MEK-ERK cascade regulates proliferation. In these cells, a downregulation of ERK correlates with a significant lower proliferation after the combination-therapy that was reflected in silico.

The in silico support a central role of MEK, AKT and Bcl2 in the apoptotic regulation in the HROC87 cells. We subsequently investigated the systemic effect of MEK or Bcl2 inhibition. The simulation of MEK inhibition led to full induction of apoptosis due to inhibition of Bcl2 even though AKT and HGFR stay active according to our experimental results (Figure 7E). Moreover, the MEK inhibition also leads to a reduction of proliferation due to the full ERK inhibition (Figure 7E). As a control of network specificity, in silico Bcl2 inhibition led to an induction of apoptosis but had no effect on proliferation (Figure 7F). Moreover, this in silico response behavior is at least supported by current clinical trials [44,45,46].

## 3. Discussion

In melanoma and non-small cell lung cancer (NSCLC) biomarker-based targeted therapies have entered the clinical routine. In this study, we try to translate these growing insights into common cancer pathways and explore the relevance of individual mutations to CRC. We introduce a tissue engineered human CRC 3D model and an in silico tool for the systemic understanding of mutational steps in cancer progression and their impact on drug efficacy that can support patient stratification.

Cell responses depend on their environment [19,34,47] as also demonstrated by other 3D methods like matrigel coating assays, spheroid cultures or models on synthetic or natural matrices [48,49]. Recently established organoid cultures maintain different cell types and enable personalized drug testing [50]. Our models aim at the detection of general interdependencies between different signaling pathways by selecting early passage cell lines with defined mutations. We have established a 3D test system for BRAF-mutant CRC using cells with different co-mutations [27,28] and show a good molecular and phenotypic reproduction of the original tumors. Advantages of our model are (I) lower proliferation similar to that in patients, (II) higher chemoresistance compared to 2D models, and (III) a comprehensive signaling profile for the tested therapies. In the last step (IV), we reflect results from 3D models by bioinformatical in silico models that connect the two most frequently impaired common signaling pathways in this CRC subgroup to allow focused target predictions.

In general, the high proliferation rates in 2D lead to an overestimation of cytostatic compounds’ efficacy [19,34]. Culturing tumor cells on a tissue matrix adjusted proliferation to in vivo levels. Furthermore, we noted in 2D models to some extent already efficacy of monotherapies, which does not reflect the clinic in CRC [9,10,51]. As we aimed at understanding network effects in a certain mutational background and at models reflecting them, we analyzed differences in central signaling cascades important for proliferation and apoptosis. Whereas in all of the three tested experimental models ERK, as a crucial protein downstream of BRAF at the end of the MAPK pathway, was inhibited in the same way by the different applied drugs, we observed differences in the central apoptosis regulator AKT. Previous studies showed that in *BRAF*-mutant cells RAF inhibition can lead to increased or unchanged AKT phosphorylation hindering thereby apoptosis induction [52]. In our 2D signaling analysis, we observed indeed an increase of AKT activation after vemurafenib mono- and combination-therapy with gefitinib, but together with apoptosis induction—contradicting former observations. Our 3D CRC models and the PDX showed only weak regulations of pAKT, going in line with no strong apoptosis induction by all therapies in 3D. Additionally, we investigated activation changes in the EGFR as important starting point of the cascades and HGFR as a receptor that is often coregulated with EGFR [53,54,55]. The EGFR showed over all models similar patterns of activation upon the different therapies. Other studies with various BRAF-mutant CRC cell lines could also show the failure of vemurafenib as monotherapy and suggest additional EGFR inhibition as a promising therapeutic approach to avoid a feedback activation of EGFR after BRAF inhibition with vemurafenib [11,56,57]. In our experiments, we did not observe a strong activation of the EGFR upon vemurafenib treatment. Interestingly, in 2D the HGFR was strongly deactivated by vemurafenib and combination-therapy, which was less prominent in 3D conditions. This could be one reason for the observed higher chemoresistance in 3D than in 2D as the activation of the HGF signaling pathway protects tumor cells from apoptosis [58], which is also reported in gefitinib-resistant cell lines [59,60]. PDX could not mirror the human in vivo situation properly because the murine HGF deriving from the tumor microenvironment did not activate the human HGFR as reported also by others [61,62]. These differences in phosphorylation patterns could explain why preclinical xenograft studies often fail in drug efficacy testing and stress the urgent need to develop fully human models.

To model CRC signaling and drug responses in specific genetic backgrounds, our in silico model integrates central cascades of commonly impaired cancer pathways [2]. We connected two common cancer pathways, i.e., MAPK and Wnt/β-catenin-pathway within our in silico topology as in the selected MSI CRC subgroup these pathways are most frequently mutated. For further improvement of patient stratification of MSI tumors with BRAF mutation as part of the MAPK pathway, we focused on alterations driving tumor progression to an invasive state across different defined intermediate states in particular on p53 in case of HROC87. Our analysis revealed in this mutational background Bcl2-Bax-BAD regulation as an important axis or even engine in the network that decides whether apoptosis is induced independently of HGFR, ERK and AKT activation. We demonstrate that in silico modeling allows to simulate targeted therapies, i.e., vemurafenib for BRAF and gefitinib for EGFR inhibition. More importantly, we can provide new suggestions in case of therapy failure, here illustrated by targeting the network-mediated activity of MEK as well as the anti-apoptotic activity of Bcl2 [63,64]. However, the Boolean logic of our in silico simulation allows the integration of several signaling cascades into the topology. This prepares the ground for possible target predictions in patient groups with frequently mutated genes in pathways such as Wnt/β-catenin, even though this was not part of the present study.

There are clinically tested combination-therapies such as ae triple combination-therapy using an EGFR inhibitor, a BRAF inhibitor as well as a MEK inhibitor. This combination is the subject of an open phase I/II study combining trametinib, dabrafenib and panitumumab [44,45] as well as an open-label, phase 3 trial with encorafenib, binimetinib, and cetuximab in BRAF V600E-mutated CRC [46]. On the other hand, venetoclax is already used as Bcl-2-specific inhibitor for chronic lymphocytic leukemia treatment [65]. This supports our therapeutic suggestion from the here established in silico model that targeting the MEK-Bcl2 axis is beneficial.

In our work, we follow the idea of tumor engines first propagated by Gerard Evans. These engines are more fundamental for tumor maintenance than drivers as they are the motor to control whole functional settings. In silico these are incorporated as network pace-makers: they are central, well-connected signaling proteins such as EGFR, KRAS, BRAF or c-Myc. Central signaling pathways induce tumor transformation by communicating with surrounding cells to generate a highly malignant tumor stroma and an immune-evasive phenotype [66]. Targeting these machineries promises longer lasting therapeutic success. The involved network checks and balances can only be appropriately modeled in sufficient complex models to understand and treat cancer-specific differences; here exemplified by the failure of therapeutic success of vemurafenib in case of BRAF mutation in CRC. We are aware that our study delivers only a proof of principle by connecting signaling, apoptosis and proliferation of one early passage cell line representing one individual patient in the in silico model. For the purpose of effective translation to the clinic further in vitro models with more cell lines harboring specific mutation signatures should be generated as well as models with primary cells available from organoid cultures. However, we tried to meet the actual demand on mode-of-action studies with this new combined in silico/in vitro tool.

## 4. Materials and Methods

### 4.1. SISmuc Preparation

SISmuc was prepared from porcine gut as previously described [31,67] followed by removal of the mesentery and the vascular tree from the completely decellularized explant. The matrix is registered under the trade mark BioVaSc-TERM^®^ (TERM, Würzburg, Germany) and after seeding with tumor cells under OncoVaSc-TERM^®^. All explantations were in compliance with the German Animal Protection Laws (§4 Abs. 3) and all animals received humane care in compliance with the guidelines by the FELASA, WHO and FDA (WHO-TRS978 Annex3 und FDA-OCTGT Preclinical Guidance) after approval from our institutional animal protection board (registration reference number #2532-2-12, Ethics Committee of the District of Unterfranken, Würzburg, Germany).

### 4.2. 2D Cell Culture and Cell Lines

We used HROC24 and HROC87 cells [29] cultured in DMEM/F12 medium supplemented with 10% FCS under standard culture conditions (37 °C, 5% CO_2_).

### 4.3. 3D Cell Culture of In Vitro Models

For static 3D cell culture, cells were seeded onto SISmuc and fixed between two metal rings as described previously [34]. All 3D cultures were performed under standard conditions (37 °C, 5% CO_2_) within DMEM/F12 medium supplemented with 10% FCS as described before [18,19]. Remarkably, we also recognized a trend in AKT activation upon combination-therapy in another FCS batch (N° BS210601.5), but also no induction of apoptosis in 3D conditions. We integrated only data from the FCS batch N° 8SB016. For monocultures, 100,000 cells in 500 µL medium were seeded onto the former luminal side of the SISmuc. After adhesion time (2 h), cell crowns with reseeded scaffolds were completely filled with medium and cultured for 14 days. On day 11 and day 13, cells were treated with 1 µM gefitinib (Iressa, Astra Zeneca, Germany) and 1 µM vemurafenib (PLX4032, S1267, Selleck Chemicals, Houston, TX, USA) as previously described [19]. Then, the tissue was washed with PBS and fixed in 4% PFA for 2h followed by paraffin embedding for immunohistochemical analysis. For WB analysis, tissue was washed twice with ice-cold PBS and lysed (30 min, 4 °C) with modified RIPA buffer. After centrifugation (14,000 rpm, 10 min, 4 °C) the supernatant of the lysate was stored (−80 °C) until use.

### 4.4. Animal Models

Male adult NMRI Foxn1nu mice with HROC87 T2 PDX in both flanks (mean volume of tumors at the beginning of therapy: 13,062 mm^3^) were treated with gefitinib (100 mg/kg body weight, i.p., 3×/week), vemurafenib (PLX4720 (kindly provided by Plexxikon, Berkeley, CA, USA) and oral, ad libitum, 417 mg/kg body weight or both, D10030403i Rodent Diet (obtained from a local distributor, Brogaarden, Denmark). A control group received 28 µL DMSO (i.p., 3×/week). Tumor volume was measured on day 0, 3, 8, 10, 14, 17 and 21 after the beginning of therapy. On day 21, mice were sacrificed; tumors were resected and fixed in 4% PFA.

### 4.5. Fluorescence Immunohistochemistry

Fixation was done with 4% PFA (glass slides: 10 min, SISmuc samples: 2 h). Glass slides were either directly stained or stored in PBS (4 °C, up to 1 week) prior to staining. SISmuc samples were stained as paraffinized sections (3–5 µm) and primary antibodies (E-cadherin (610181, BD Bioscience, San Jose, CA, USA), β -catenin (ab32572, Abcam, Cambridge, UK), vimentin (ab92547, Abcam), PCK (C2562, Sigma Aldrich, St. Louis, MO, USA), Ki67 (ab16667, Abcam) were diluted 1:100 in 5% BSA in PBS (w/v) and incubated overnight (4 °C). Secondary antibodies were diluted 1:400 and incubated for 1h at room temperature (RT). Double staining were performed using primary antibodies of two different species and secondary antibodies marked with fluorescent dyes (Alexa-647, Alexa-555, Life Technologies, Carlsbad, CA, USA). Nuclei were counterstained (DAPI in Mowiol embedding solution). Pictures were taken using a confocal laser scanning microscope (SP-8, Leica, Wetzlar, Germany) or a digital microscope (BZ-9000, Keyence, Neu-Isenburg, Germany).

### 4.6. Total Cell Number and Proliferation Rate

To determine the total cell number per image (20× magnification), all DAPI-positive cells were evaluated. Ki67-positive cells were counted and presented as percentage of the total cell number per image.

### 4.7. Protein Lysate Preparation and WB Analysis

Cells on SISmuc or in Petri dishes were washed twice with ice-cold PBS and lysed (30 min, 4 °C) on a rocking platform with modified RIPA buffer containing 137 mM NaCl, 50 mM NaF, 20 mM Tris (pH 8), 2 mM EDTA, 10% (v/v) glycerol, 1.0% (v/v) NP40, 0.5% (w/v) sodium deoxycholate, 0.1% (w/v) SDS, and freshly added 1 mM Na3VO4 and protease inhibitor cocktail tablets (cOmplete™ mini EDTA-free, Roche, Basel, Switzerland). The lysate was centrifuged (14,000 rpm, 10 min, 4 °C) and the supernatant transferred into clean tubes for quantitation of the protein concentration using DC Protein Assay (500-0116, BioRad, Hercules, CA, USA) or stored (−80 °C) until further use. Before SDS gel electrophoresis, a protein precipitation with methanol/chloroform according to Wessel and Flügge [68], was performed. Antibodies used for WB: EGFR (4267, CST), p-EGFR (Tyr1068, Y68) (32430, Abcam), Met (8198, CST), p-Met (Tyr1234/1235) (3077, CST, Danvers, MA, USA), Erk1/2 (05-1152, Millipore, Burlington, MA, USA), p-Erk1/2 (Thr202/Tyr204) (4370, CST), Akt (pan) (4691, CST), p-Akt (Ser473) (4060, CST), α-Tubulin (3873, CST). We used the Protein Ladder_ProSieve QuadColor Protein Marker (Lonza, Basel, Switzerland).

### 4.8. M30 CytoDeathTM ELISA

M30 CytoDeath™ ELISA (10900, Tecomedical, Bünde, Germany) is used for the quantitative determination of the apoptosis-associated K18Asp396 (M30) neo-epitope in cultured human, monkey or bovine cells. The M30 antibody recognizes caspase-cleaved cytokeratin 18 (ccK18). Cell culture medium supernatants were collected 24h before treatment as well as 24 h, 48 h and 72 h after treatment and stored (−80 °C) until measurement. M30 CytoDeath™ ELISA was performed according to the manufacturer’s instructions.

### 4.9. Statistical Analysis

All statistical analysis was performed with the open-source software R [69]. Differences with a value of *p* < 0.05 were considered statistically significant. All experiments were performed at least in triplicates and the results presented as boxplots; statistical significance was calculated by Student’s *t*-test.

### 4.10. In Silico Simulations

For generation of the CRC in silico topology, we used CellDesigner (Version 3.5). For semi-quantitative analyses, we used SQUAD [43]. The in silico topology was created on the basis of literature and databank information followed by simulations of 3D in vitro data. The appendix provides details on network setup of the colon cell-specific model and in silico simulation of different therapies for HROC87 cells.

## 5. Conclusions

We consider a systemic view on targeted treatment approaches as well as so-called tumor engines of CRC instead of isolated drivers and non-relevant or bystander alterations. Furthermore, signaling in cancer cells depends on the cells’ environment and cannot be reflected accurately by oversimplified 2D culture or animal experiments due to species differences in signaling cascades such as HGFR. We meet these challenges by the identification of cellular network changes in the functional context of refined 3D tissue models mirroring better clinical responses and by a deeper understanding of common signaling alterations in CRC and its specific subtypes in silico that can be translated to the clinic by improving patient stratification.

## Figures and Tables

**Table 1:** Pre-stimulation activities of the in silico model as starting point for the therapy simulation.

Name of Network Node	untr.	+gef	+vem	+combi	+MEK-Inh	+BCL2-Inh
BRAF(V600)-Act	0.7	0.7	0.7	0.7	0.7	0.7
EGFR	0.5	0.5	0.5	0.5	0.5	0.5
HGFR	0.7	0.7	0.7	0.7	0.7	0.7
ERK-Inh	0.26	0.3	0.3	0.41	0.26	0.26
p53-Mut	0.2	0.2	0.2	0.2	0.2	0.2
Bcl2	0.31	0.3467	0.305	0.2252		
c-Myc-Inh	0.17	0.097	0.097		0.17	0.17
AKT	0.2	0.16	0.2	0.24	0.2	0.2
(EGFR) *		0.3	0.4	0.3		
(HGFR) *		0.6		0.4		
c-Myc-Act				0.09		
Gefitinib		1.0		1.0		
Vemurafenib			1.0	1.0		
MEK-Inhibitor					1.0	
Bcl2-Inhibitor						1.0

* denotes that the protein for which this node stands for is the activated form of the receptor.

**Table 2:** Simulation outcome of the pre-stimulation settings ^1^ to mimic the cell culture response behavior of HROC87 cells.

Name of Network Node	untr.	+gef	+vem	+combi	+MEK-Inh	+BCL2-Inh
apoptosis	0.21	0.35	0.35	0.35	0.99	0.99
proliferation	0.5	0.5	0.5	0.2	0.0	0.5
(HGFR)*	0.97	0.6	0.97	0.4	0.97	0.97
ERK	0.71	0.59	0.59	0.3	0.0	0.71
(EGFR)*	0.5	0.3	0.4	0.3	0.5	0.5
AKT	0.2	0.16	0.2	0.24	0.2	0.2

^1^ perturbation function of SQUAD, pre-stimulations in Table 1. * denotes that the protein for which this node stands for is the activated form of the receptor.

## Appendix A

For all experiments we used PDX generated from a patient tumor (of patient HROC87) in passage 3 in immunodeficient mice.

**Table A1:** Grey values (in % of control) of the representative blot in Figure 5.

**2D**								
Figure 5	EGFR	Erk	pEGFR	pErk	HGFR	Akt	pHGFR	pAkt
Ctrl.	100	100	100	100	100	100	100	100
G	70	96	49	32	44	76	1	227
V	75	77	80	18	52	73	86	130
GV	63	94	53	2	26	86	2	314
**3D**								
Figure 5	EGFR	Erk	pEGFR	pErk	HGFR	Akt	pHGFR	pAkt
Ctrl.	100	100	100	100	100	100	100	100
G	111	128	36	40	91	114	67	74
V	81	83	86	7	72	65	78	75
GV	55	91	61	2	55	70	34	93
**PDX**								
Figure 5	EGFR	Erk	pEGFR	pErk	HGFR	Akt	pHGFR	pAkt
Ctrl.	100	100	100	100	100	100	-	100
G	70	102	37	38	55	134	-	27
V	53	67	32	10	55	88	-	17
GV	77	104	22	4	55	89	-	18

### Appendix A.1. Ideal-Typic Transformation from a Healthy Epithelium to a Colon Carcinoma

Transformation to malignant stages usually happens step-wise over time involving different activation processes (Table A2). Here, we give a simplified example and route of the suppressor pathway. In a first step of cancer progression, the β-catenin signaling pathway is changed by a mutation. In 85% of all early stages, APC is changed by mutation which leads to increased cell proliferation [69,70]. Therefore, the normal epithelium (stage 0) transforms by dysplasia to APC (stage 1). An aberration of COX-2 then leads to the early adenoma stage (stage 2) [71]. Afterwards, mutations of KRAS lead to the intermediate adenoma (stage 3), detectable in 55 % of all cases [72]. The late adenoma (stage 4) is based on loss of 18q / DCC [38]. Mutations in the p53 gene lead to the carcinoma (stage 5) [38]. Metastasis (stage 6) is induced possibly via E-cadherin, the activation of molecules involved in the TGFβ signaling pathway (Smad2, Smad4, TGFβR). Further tumor progression selects for more resistant cells and may include activation of antiapoptotic pathways e.g., by the protein BAX [73,74].

**Table A2:** Stepwise transformation of a normal colon epithelial cell to colon carcinoma.

Stage 0	Stage 1	Stage 2	Stage 3	Stage 4	Stage 5	Stage 6
normal epithelium	dysplastic aberrant cryptic foci	early/initial adenoma	intermediate adenoma	late adenoma	carcinoma	metastasis
	*APC* (β-*catenin*)	*COX-2*	*KRAS*	*DCC*/loss of 18q	*p53*	*E-cadherin*/*BAX*

### Appendix A.2. Bioinformatics Mod$eling and Systems Biology Data

Bioinformatics modeling of the in vitro data-uses data-driven modeling based on Boolean semi-quantitative models. These start from (i) a Boolean model (yes and no decisions) of the network and then (ii) approximates by exponential functions the behavior of the individual kinases, pathways and cellular reactions (e.g., apoptosis, growth, differentiation) over time.

(i) We started our modeling by setting-up a simplified network model of the colon cancer cell using literature (see also Table A2) and databases. The model includes three parts: (a) central tumor signaling cascades such as MAPK and its connection to apoptosis and proliferation, (b) tumor-specific mutations (HROC87: BRAF, p53) and experimental measured nodes (experimental data summarized in Table A3) and (c) the treatments.

The network was visualized using CellDesigner Software (Figure A8). (ii) Next, the network model was step-wise iteratively refined looking at the experimental data of cell culture treatment and cellular reaction as well as the specific modifications to closely correspond to the behavior of the HROC87 tumor cells in cell culture regarding apoptosis, proliferation and signaling (in particular its specific mutations as well as different reactivity to different drugs). This was modeled by basic pre-stimulation (activation, inhibition) of the pathways and specific nodes as we only focus on the central part of the cellular network involving not all modulatory nodes available in the cell (Table 1). The pre-stimulation mimics hence stronger or lower responsiveness of the tumor pathways. The stimulation settings were step-wise optimized to follow as close as possible the experimental data and taking note of the Boolean network topology logic of the signaling pathways as shown in Figure A8.

**Table A3:** Pre-stimulation activities of the in silico model as starting point for the therapy simulation.

Western Blot Data (in Comparison to Untreated Control)					Proliferation Value in 3D			Apoptosis in 3D Compared to Ctrl	
	Ctrl	Gef	Vem	Gef+Vem			HROC87		HROC87
pEGFR	medium	↓↓	↓	↓↓	Ctrl		0.5	Ctrl	weak
pErk	strong	↓	↓	↓↓	Gef		0.5	Gef	↑
pHGFR	strong	↓	0	↓↓	Vem		0.5	Vem	↑
pAkt	weak	↓	0	↑	Gef + Vem		0.2	Gef+Vem	↑

The pre-stimulation was done using the perturbation function in SQUAD. The simulations are shown in Figure 7 and in Figure A9 for vemurafenib treatment. The activation outcomes after the pre-stimulation settings are listed in Table 2, reflecting the cell culture response behavior of the HROC87 cells from Table A3.

### Appendix A.3. Simulation of Different Treatment Scenarios

Several treatment in silico simulations are already given in the results section, we show here additionally the simulation of vemurafenib treatment.
